# The asialoglycoprotein receptor in human hepatocellular carcinomas: its expression on proliferating cells

**DOI:** 10.1038/sj.bjc.6690708

**Published:** 1999-10

**Authors:** D Trerè, L Fiume, L Badiali De Giorgi, G Di Stefano, M Migaldi, M Derenzini

**Affiliations:** Servizio di Citopatologia, Policlinico S Orsola, Bologna, Italy; Dipartimento di Patologia Sperimentale, Università di Bologna, Bologna, Italy; Dipartimento di Scienze Morfologiche e Medico Legali, Sezione di Anatomia Patologica, Università di Modena, Modena, Italy

**Keywords:** immunocytochemistry, drug targeting, bromodeoxyuridine, S phase

## Abstract

The expression of the asialoglycoprotein receptor (ASGP-R) on human hepatocellular carcinoma (HCC) cells might be exploited to reduce the extrahepatic toxicity of DNA synthesis inhibitors by their conjugation with galactosyl-terminating peptides. In the present study we first assessed the frequency of ASGP-R expression in 60 HCCs. Secondly, we investigated whether the receptor was maintained on the plasma membranes of DNA synthesizing cancer cells. Needle biopsies of HCC were evaluated. Diagnosis and grading of HCC were performed on routine haematoxylin and eosin-stained sections according to Edmondson and Steiner (1953). Thirty-five tumours were grade I and II and were classified as well differentiated, while 25 tumours were grade III and IV and were classified as poorly differentiated. Sections from formalin-fixed, paraffin-embedded samples were incubated, after antigen retrieval, with an anti-ASGP-R monoclonal antibody revealed by secondary biotinylated antibody and streptavidin–biotin–peroxidase–diaminobenzidine reaction. A clear immunolabelling of plasma membranes of HCC cells was observed in 28 out of 35 (80%) well differentiated (grade I and II) and in five out of 25 (20%) poorly differentiated (grade III and IV) HCCs. The presence of the ASGP-R on the surface of DNA synthesizing cancer cells was also investigated after in vitro bromodeoxyuridine (BrdU) labelling of HCC samples by immunohistochemical visualization of both the ASGP-R and incorporated BrdU on the same section. The results obtained clearly demonstrated that DNA synthesizing cancer cells expressed the ASGP-R on their surface. The presence of ASGP-R on cell plasma membrane in the majority of differentiated HCCs and its maintenance on proliferating cells encourages studies in order to restrict the action of the inhibitors of DNA synthesis of HCC cells by their conjugation with galactosyl-terminating carriers internalized through this receptor. © 1999 Cancer Research Campaign


(1999) **81**(3), 404–408
© 1999 Cancer Research Campaign
Article no. bjoc.1999.0708

### The asialoglycoprotein receptor in human hepatocellular carcinomas: its expression on proliferating cells

**D Trerè[1,2], L Fiume[2], L Badiali De Giorgi[1], G Di Stefano[2], M Migaldi[3] and M Derenzini[1,2]**

[1]Servizio di Citopatologia, Policlinico S Orsola, Bologna, Italy; [2]Dipartimento di Patologia Sperimentale, Università di Bologna, Bologna, Italy; [3]Dipartimento di Scienze Morfologiche e Medico Legali, Sezione di Anatomia Patologica, Università di Modena, Modena, Italy

**Summary** The expression of the asialoglycoprotein receptor (ASGP-R) on human hepatocellular carcinoma (HCC) cells might be exploited to reduce the extrahepatic toxicity of DNA synthesis inhibitors by their conjugation with galactosyl-terminating peptides. In the present study we first assessed the frequency of ASGP-R expression in 60 HCCs. Secondly, we investigated whether the receptor was maintained on the plasma membranes of DNA synthesizing cancer cells. Needle biopsies of HCC were evaluated. Diagnosis and grading of HCC were performed on routine haematoxylin and eosin-stained sections according to Edmondson and Steiner (1953). Thirty-five tumours were grade I and II and were classified as well differentiated, while 25 tumours were grade III and IV and were classified as poorly differentiated. Sections from formalin-fixed, paraffin-embedded samples were incubated, after antigen retrieval, with an anti-ASGP-R monoclonal antibody revealed by secondary biotinylated antibody and streptavidin–biotin–peroxidase–diaminobenzidine reaction. A clear immunolabelling of plasma membranes of HCC cells was observed in 28 out of 35 (80%) well differentiated (grade I and II) and in five out of 25 (20%) poorly differentiated (grade III and IV) HCCs. The presence of the ASGP-R on the surface of DNA synthesizing cancer cells was also investigated after in vitro bromodeoxyuridine (BrdU) labelling of HCC samples by immunohistochemical visualization of both the ASGP-R and incorporated BrdU on the same section. The results obtained clearly demonstrated that DNA synthesizing cancer cells expressed the ASGP-R on their surface. The presence of ASGP-R on cell plasma membrane in the majority of differentiated HCCs and its maintenance on proliferating cells encourages studies in order to restrict the action of the inhibitors of DNA synthesis of HCC cells by their conjugation with galactosyl-terminating carriers internalized through this receptor. © 1999 Cancer Research Campaign

**Keywords:** immunocytochemistry; drug targeting; bromodeoxyuridine; S phase

The asialoglycoprotein receptor (ASGP-R) is a glycoprotein present in large amount only on hepatocytes where it is expressed on the sinusoidal and lateral plasma membrane (Morell et al, 1968; Geffen and Spiess, 1992). ASGP-R binds and internalizes a broad range of molecules exposing galactose or *N*-acetyl-galactosamine residues. Following internalization the fate of ligand is lysosomal degradation (Morell et al, 1968; Geffen and Spiess, 1992).

Taking advantage of this receptor a chemotherapeutic approach has been developed to reduce the extrahepatic side-effects of nucleoside analogues (NAs) used in chronic viral hepatitis (Fiume et al, 1979, 1997; Torriani et al, 1996). These drugs are coupled to galactosyl-terminating peptides. The conjugates selectively enter hepatocytes, where the lysosomal enzymes split the bond between the carrier and the drug, which becomes concentrated in liver cells. A similar strategy was suggested in order to increase the chemotherapeutic index of drugs inhibiting DNA synthesis in the treatment of human hepatocellular carcinoma (HCC) (Schneider et al, 1984; O'Hare et al, 1989; Di Stefano et al, 1998). This approach obviously requires the presence of the receptor in the neoplastic tissue and maintenance of its expression on DNA synthesizing cells.

Few and conflicting data are available on the distribution of ASGP-R in human neoplastic hepatocytes. In a study in which the receptor was measured by a biochemical procedure, it was not found in HCC samples (Sawamura et al, 1984), whereas in an immunohistochemical investigation carried out on ten cases of HCC the presence of the receptor was demonstrated in the more differentiated forms (Hyodo et al, 1993). In the present study we have first assessed the frequency of ASGP-R expression in a large number of human HCCs. For this purpose, needle biopsy samples of sixty consecutive HCCs were analysed. Visualization of the receptor was obtained using an anti-ASGP-R monoclonal antibody applied after antigen retrieval procedure to routinely formalin-fixed, paraffin-embedded liver samples. Since we found that the ASGP-R was present in 33 HCCs we also investigated whether the ASGP-R was maintained on the plasma membrane of DNA synthesizing cancer cells. For this purpose we incubated HCC tissue samples with bromodeoxyuridine (BrdU). The presence of ASGP-R on DNA-synthesizing cancer cells was immunohistochemically assessed using anti-BrdU and anti-ASGP-R antibodies on the same tissue section.

*Received 11 August 1998*
*Revised 22 December 1998*
*Accepted 22 December 1998*

*Correspondence to:* M Derenzini, Università degli Studi di Bologna, Servizio di Citopatologia, Policlinico S Orsola, Via Massarenti 9, 40138 Bologna, Italy

### MATERIALS AND METHODS

### Specimens

A surgically resected liver tissue sample adjacent to metastasis of colonic adenocarcinoma was fixed in 10% neutral-buffered formalin and routinely paraffin-embedded.

Needle biopsies from 60 consecutive cases of HCCs from the archives of the Department of Morphological Sciences and Legal Medicine, Section of Anatomic Pathology, of Modena University were evaluated. This series included only primary HCCs. Samples had been fixed in 10% neutral-buffered formalin and routinely paraffin-embedded. Diagnosis and grading of HCC were performed on routine haematoxylin and eosin (H&E)-stained sections according to Edmondson and Steiner (1953).

Surgically resected liver samples from three patients with primary HCC were processed for in vitro BrdU labelling as described by Trerè et al (1991). Briefly, immediately after surgical resection the histological sample was divided into small pieces with a maximum diameter of 0.5–1.0 mm which were then immersed in 5 ml of RPMI-1640 containing non-essential amino acids (Seromed, Biochrom KG), 100 U $ml^{-1}$ penicillin, 100 μg $ml^{-1}$ streptomycin and 10% fetal calf serum (FCS) supplemented with 160 μM BrdU (Sigma Co., USA), and incubated for 4 h at 37°C in a 5% carbon dioxide–air incubator. Tissues were then rapidly rinsed with three changes of cold phosphate-buffered saline (PBS) (0.01 M, pH 7.5) and fixed in 70% ethanol for 48 h at 4°C. Finally, tissues were routinely processed and embedded in paraffin.

### Patients

Forty-four men and 16 women were studied, with a mean age of 63 ± 12 years. Serum markers for hepatitis B virus (HBV), anti-HCV antibodies and α-fetoprotein (AFP) were assessed by commercial kits. Fifty-four patients had cirrhosis. This was related to alcohol abuse alone in five, alcohol abuse combined with chronic hepatitis C in two, HBV infection in 14 and HCV infection in 31. No risk factors were recognized in two cirrhotic patients. The remaining six patients included four with chronic hepatitis B, one with chronic hepatitis C and one with histologically normal liver. Tumour size was evaluated at the time of HCC diagnosis by ultrasound examination.

### ASGP-R immunolabelling

From each paraffin block, 3- to 4-μm-thick sections were cut, cleared in xylene and rehydrated. Slides were then removed from water to plastic slide holders, fully immersed in 10 mM sodium citrate buffer (pH 6.0) and heated for 20 min at 120°C in a commercially available pressure cooker. After cooling to room temperature in the sodium citrate buffer, slides were treated with a solution of 0.3% hydrogen peroxide ($H_2O_2$) in methanol for 30 min at room temperature to abolish endogenous peroxidase activity. Sections were then incubated for 10 min in a moist chamber with non-immune rabbit serum diluted to 5% in PBS pH 7.2–7.4 to reduce non-specific background staining. Sections were then incubated overnight at 4°C with the primary antibody (anti-ASGP-R monoclonal antibody, purchased from Daiichi Pure Chemicals Co. Ltd, Tokyo, Japan) diluted 1:200. This antibody was produced by Kohgo et al (1993) by immunizing Balb-c mice with the ASGP-R purified from human liver. Sections were subsequently incubated with rabbit anti-mouse Ig biotinylated antibody (Dako, Glostrup, Denmark) diluted 1:600 in PBS with 5% normal human AB serum for 30 min, and then in a streptavidin–biotin–peroxidase preformed complex (Dako, Glostrup, Denmark) for 30 min. The immunologic reaction was developed using 3,3′-diaminobenzidine-tetra-hydro-chloride-$H_2O_2$ solution, dehydrated and mounted with Canada balsam (Merck, Darmstadt, Germany). A negative control was performed by omitting the anti-ASGP-R monoclonal antibody.

### Double immunolabelling for ASGP-R and BrdU

From the paraffin-embedded small tissue samples, which had been incubated in vitro with BrdU, 3- to 4-μm-thick sections were cut, cleared in xylene and rehydrated, through graded ethanol to distilled water. Slides were placed in plastic slide holders and heated for 20 min at 120°C as previously described. After cooling in the sodium citrate buffer, slides were treated with a solution of 0.3% $H_2O_2$ in methanol for 30 min to abolish endogenous peroxidase activity, and treated with 4 N hydrochloric acid at room temperature for 20 min. Sections were then incubated in a moist chamber with non-immune rabbit serum diluted to 5% in PBS pH 7.2–7.4 for 10 min to reduce non-specific background staining, and then incubated with an anti-BrdU monoclonal antibody diluted 1:500 (Becton Dickinson, USA) for 1 h at room temperature. After three washes in PBS (10 min each), sections were incubated overnight at 4°C with the anti-ASGP-R monoclonal antibody diluted 1:200. The immunoreaction was then developed as previously described. For each experiment, three negative controls were performed by omitting the anti-ASGP-R monoclonal antibody (control I), the anti-BrdU monoclonal antibody (control II) and both monoclonal antibodies (control III) respectively.

### Statistical analysis

Differences between groups were evaluated by the ANOVA test. A *P*-value lesser than 0.05 was considered to be significant.

### RESULTS

### Clinical and histopathological characteristics HCCs

AFP levels ranged from 1.6 to 1690 ng $ml^{-1}$, with a mean value of 204.4 ± 382.3 ng $ml^{-1}$. The maximum diameter of HCCs ranged from 1.5 to 12 cm, with a mean value of 4.34 ± 2.60 cm.

Histological grade of HCCs was assessed according to Edmondson and Steiner (1953). Thirty-five tumours were grade I and II and were classified as well differentiated. Twenty-five tumours were grade III and IV and were classified as poorly differentiated. Two out of the three surgically resected HCCs processed for in vitro BrdU labelling were grade II and one was grade III.

### Expression of the ASGP-R on human normal hepatocytes

Immunohistochemical staining of ASGP-R in formalin-fixed, paraffin-embedded human liver tissue adjacent to metastasis of a colonic adenocarcinoma revealed oxidized diaminobenzidine deposits mainly located on the sinusoidal margins of the liver

© 1999 Cancer Research Campaign

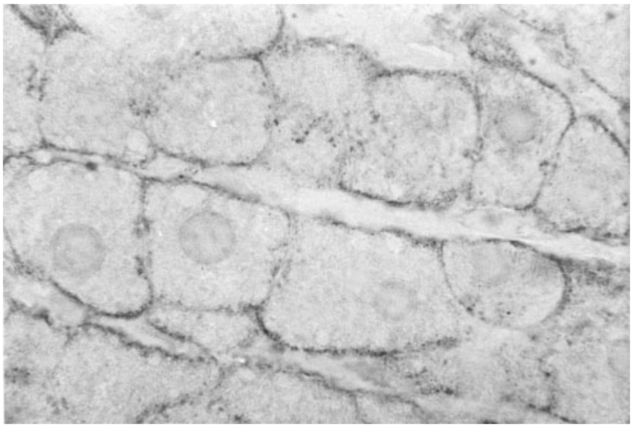

**Figure 1–3** Sections treated with anti ASGP-R monoclonal antibody revealed by peroxidase–diaminobenzidine reaction.
**Figure 1** Normal liver. The staining reaction is exclusively confined to the luminal and lateral plasma membranes of the hepatocytes. No other cellular structures are stained (× 1200)

cords. Occasionally, the immunoreaction was also visible on the lateral plasma membrane, where the quantity of reaction product was always lower. The staining reaction appeared to be uniformly distributed throughout the three acinal zones. In normal liver specimens no immunolabelling on the cytoplasm of the hepatocytes was observed (Figure 1). If the anti-ASGP-R monoclonal antibody was omitted from the immunostaining procedure, plasma membranes were not labelled.

### Expression of the ASGP-R on human HCCs

HCCs were judged as positive for ASGP-R expression when all the cancer cells exhibited clear-cut staining of the plasma membranes (Figure 2). Immunostaining of the cancer cell plasma membranes was clearly observed in 28 out of the 35 (80%) well differentiated and in five out of the 25 (20%) poorly differentiated HCCs. The intensity of the staining reaction was not to the same degree in the 33 positive HCCs. A loss of the polarized location of ASGP-R, typical of the normal hepatocytes (Geffen and Spiess, 1992), appeared to occur in the positive HCCs. Cancer cells were delimited by a uniformly stained plasma membrane (Figure 3). In seven out of the 35 well differentiated, and in 20 out of the 25 poorly differentiated HCCs no ASGP-R immunostaining was detected. Only a very light, unspecific staining of all cellular components was observed. In some cases a granular immunolabelling was detected throughout the cytoplasm of cancer cells, regardless of the presence of plasma membrane immunopositivity.

In all biopsies showing non-tumour areas adjacent to HCC, normal hepatocytes were found to be positive for ASGP-R immunostaining, independently of ASGP-R expression of cancer cells. No significant differences were found in the AFP levels between ASGP-R positive and negative patients, and no correlation was demonstrated between ASGP-R expression and aetiology. The maximum diameters of HCCs which did not present the ASGP-R were larger than those of HCCs exhibiting the ASGPR (5.10 ± 2.49 and 3.63 ± 2.54 cm respectively; F = 4.635; $P$ = 0.036). However, the correlation between ASGP-R expression and tumour size was due to the fact that well differentiated HCCs were significantly greater than poorly differentiated HCCs (with

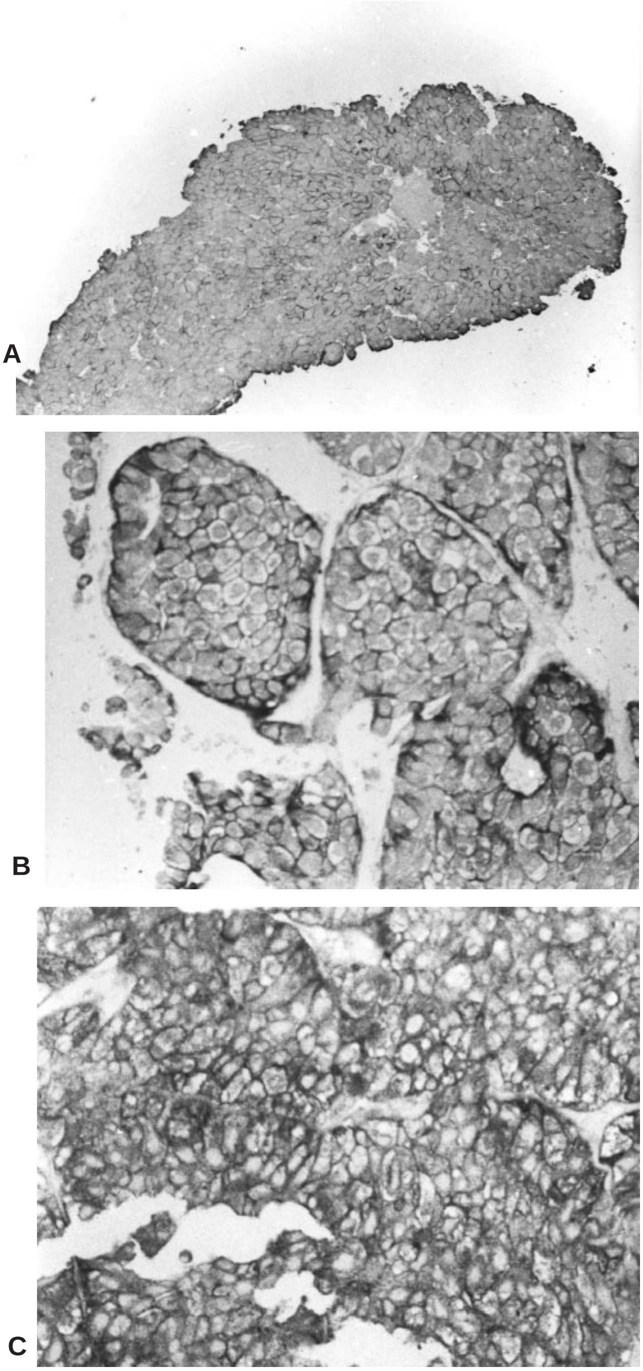

**Figure 2** Well differentiated HCCs. All HCC cells exhibit a selective immunostaining of plasma membranes (**A**, × 150; **B** and **C**, × 350)

maximum diameters of 5.15 ± 3.01 and 3.69 ± 2.04 cm respectively F = 4.488; $P$ = 0.039).

### Expression of the ASGP-R on DNA synthesizing cancer cells

When ASGP-R was immunohistochemically assessed in the three surgically resected HCCs processed for in vitro BrdU labelling, it was found to be expressed on the plasma membranes of the two grade II tumours, while the grade III tumour did not show any

© 1999 Cancer Research Campaign

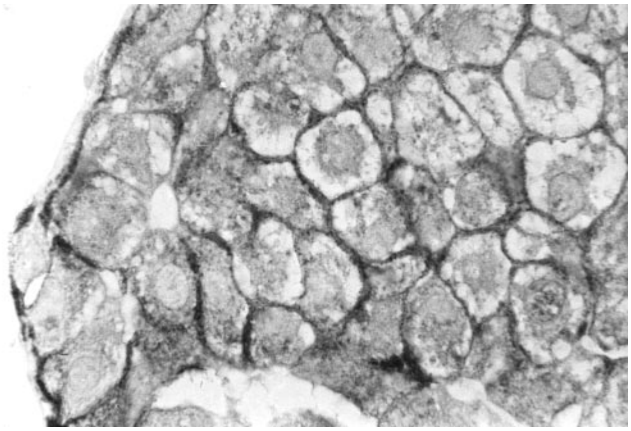

**Figure 3** Same sample as in Figure 2A shown at higher magnification (× 1200). The plasma membranes of HCC cells are uniformly stained by the immunolabelling

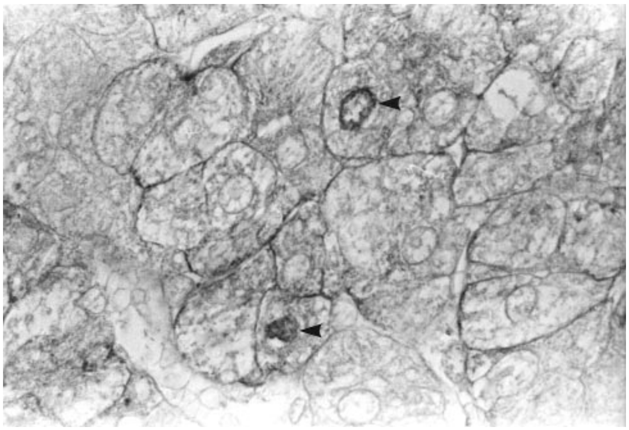

**Figure 4** Well differentiated HCC. The sample was labelled in vitro with BrdU. The section was immunostained for both BrdU and ASGP-R. Two cancer cell nuclei are stained (arrowheads); the plasma membrane of these cells is stained as well as that of the other cancer cells (× 1200)

immunostaining. A similar ASGP-R immunostaining was observed in the corresponding tumour samples routinely formalin-fixed and paraffin-embedded. The BrdU labelling indices of these HCCs, expressed as per cent labelled nuclei over at least 2000 cells counted directly at the microscope, were 7.2 and 8.1 in the two Grade II tumours respectively, and 6.6 in the grade III tumour.

To demonstrate the contemporary presence of BrdU-positive nuclei and ASGP-R expression in the same cell, visualization of both BrdU and ASGP-R was carried out on the same tissue section from the two HCCs expressing the ASGP-R out of the three HCCs pre-treated by in vitro incubation with BrdU. All BrdU-positive cells also exhibited an evident ASGP-R expression. In Figure 4 cancer cells in which the plasma membranes are clearly stained by anti-ASGP-R monoclonal antibody are shown: in two cells clear immunopositivity for BrdU is also observed in the nuclei.

### DISCUSSION

The present study, carried out on 60 consecutive human HCCs, demonstrated that the ASGP-R, as revealed using a specific monoclonal antibody, was present in 28 out of 35 well differentiated (grade I and II) and in five out of 25 poorly differentiated (grade III and IV) HCCs. The ASGP-R was immunohistochemically visualized in routinely formalin-fixed, paraffin-embedded tissues samples after the antigen retrieval procedure. We have also observed that in ASGP-R-positive HCCs the immunostaining reaction decorated all the cells composing the neoplastic tissue. This finding suggested that also the proliferating cells of the cancer lesions should express the ASGP-R on their surface. To get a direct evidence that dividing human hepatocarcinoma cells express the ASGP-R we in vitro labelled HCC samples with BrdU and immunocytochemically visualized both the ASGP-R and incorporated BrdU on the same tissue section. The results obtained clearly demonstrated that DNA synthesizing cells maintained the ASGP-R on their plasma membranes.

Our data are consistent with and extended the observations of Hyodo et al (1993) who examined ten cases of HCCs characterized by various differentiation degree and observed that ASGP-R was expressed in four of the six differentiated forms. However, no evidence was obtained by these authors that the receptor was maintained on the proliferating neoplastic cells.

The presence of ASGP-R on cell plasma membrane in the majority of differentiated HCCs and its expression on proliferating cells might be exploited to improve the chemotherapy of these tumours by conjugation of inhibitors of DNA synthesis with galactosyl-terminating carriers. In patients with HCC who are not eligible for surgical treatment or liver transplantation, chemotherapy either by systemic injection or transcatheter arterial embolization with anti-tumour agents is used (Bruix, 1997). However, systemic chemotherapy is characterized by a very low response rate with extrahepatic tissue toxicity (Bruix, 1997) and arterial chemo-embolization, even if it increases the local concentration of drugs and reduces systemic side-effects, is somewhat traumatic, responsible for the painful embolization syndrome and may favour pulmonary metastasis (Boix et al, 1996). All these drawbacks might be avoided or reduced by a chemotherapeutic approach based on the conjugation of anti-proliferating drugs specifically inhibiting DNA synthesis with carriers which bind to the ASGP-R. This treatment should kill proliferating hepatocarcinoma cells without affecting normal resting hepatocytes and the proliferating cells of other tissues which do not possess the ASGP-R. Obviously, the prerequisite for this chemotherapeutical approach is the immunocytochemical detection of the ASGP-R in the neoplastic specimens obtained by needle biopsy. Only for these tumours, the treatment with a conjugated drug could be advantageous.

The presence of the receptor on the surface of proliferating cancer cells does not assure that the conjugated drug after the ASGP-R binding is internalized and released from the carrier by lysosomal enzymes as in normal resting hepatocytes. However, in recent experiments evidence was obtained that this process can also occur in neoplastic hepatocytes. Using Hep G2 cells, a human HCC cell line expressing the ASGP-R (Schwartz et al, 1981), it was found that 5-fluoro-2′-deoxyuridine conjugated with lactosaminated poly-L-lysine inhibited cell proliferation after intracellular penetration through the receptor (Di Stefano et al, 1998). This result, together with the present observations, suggest conjugation with galactosyl-terminating carriers as a possible way to restrict the pharmacological action of DNA synthesis inhibitors to HCC cells and prevent their side-effects on extrahepatic tissues.

*© 1999 Cancer Research Campaign*

### ACKNOWLEDGEMENTS

This work was supported by grants from Ministero della Ricerca Scientifica e Tecnologica (MURST) 40 and 60%, Pallotti's Legacy for Cancer Research, Associazione Italiana per la Ricerca sul Cancro (AIRC Milan, Italy), and the University of Bologna (funds for selected research topics).

### REFERENCES

Boix L, Briux J, Castells A, Vianney A, Llovet JM, Rivera F and Rodes J (1996) Circulating mRNA for alpha-fetoprotein in patients with hepatocellular carcinoma. Evidence of tumor dissemination after trans arterial embolization [Abstract]. *Hepatology* **24**: 349A

Bruix J (1997) Treatment of hepatocellular carcinoma. *Hepatology* **25**: 259–262

Di Stefano G, Busi C, Derenzini M, Trerè D and Fiume L (1998) Conjugation of 5-fluoro-2′-deoxyuridine with lactosaminated poly-L-lysine to reduce extrahepatic toxicity in the treatment of hepatocarcinomas. *Ital J Gastroenterol Hepatol* **30**: 173–177

Edmondson HA and Steiner PE (1953) Primary carcinoma of the liver: a study on 100 cases among 489 000 necropsies. *Cancer* **7**: 462–503

Fiume L, Di Stefano G, Busi C, Mattioli A, Bonino F, Torrani Cerenzia M, Verme G, Rapicetta M, Bertini M and Gervasi GB (1997) Liver targeting of antiviral nucleoside analogues through the asialoglycoprotein receptor. *J Viral Hepatol* **4**: 363–370

Fiume L, Mattioli A, Balboni PG, Tognon M, Barbanti Brodano G, De Vies J and Wieland T (1979) Enhanced inhibition of virus DNA synthesis in hepatocytes by trifluorothymidine coupled to asialofetuin. *FEBS Lett* **103**: 47–51

Geffen I and Spiess M (1992) Asialoglycoprotein receptor. *Int Rev Cytol* **137**: 181–219

Hyodo I, Mizuno M, Yamada G and Tsuji T (1993) Distribution of asialoglycoprotein receptor in human hepatocellular carcinoma. *Liver* **13**: 80–85

Kohgo Y, Kato J, Nakaya R, Mogi Y, Yago H, Sakai Y, Matsushita H and Niitsu Y (1993) Production and characterization of specific asialoglycoprotein receptor antibodies. *Hybridoma* **12**: 591–598

Morell AG, Irvine RA, Sternlieb I, Scheinberg IH and Ashwell G (1968) Physical and chemical studies on ceruloplasmin. V. Metabolic studies on the sialic acid-free ceruloplasmin in vivo. *J Biol Chem* **243**: 155–159

O'hare KB, Hume IC, Scarlett L, Chytrý V, Kopeckovà P, Kopecek J and Duncan R (1989) Effect of galactose on interaction of N-(2-hydroxypropyl) methacrylamide copolymers with hepatoma cells in culture: preliminary application to an anticancer agent, daunomycin. *Hepatology* **10**: 207–214

Sawamura T, Naka H, Hazama H, Shiozaki Y, Sameshima Y and Tashiro Y (1984) Hyperasialoglycoproteinemia in patients with chronic liver diseases and/or liver carcinoma. Asialoglycoprotein receptor in cirrhosis and liver cell carcinoma. *Gastroenterology* **87**: 1217–1221

Schneider YJ, Abarca J, Aboud-Pirak E, Baurain R, Ceulemans F, Deprez De Campeneere D, Lesur B, Masquelier M, Otte Slachmuylde C, Rolin Van Swieten D and Trouet A (1984) Drug targeting in human cancer chemotherapy. In: *Receptor-Mediated Targeting of Drugs*, Gregoriadis G, Poste G, Senior J and Trouet A (eds), pp. 1–25. NATO ASI Series A: Life Sciences. Plenum Press: New York

Schwartz AL, Fridovich SE, Knowles BB and Lodish HF (1981) Characterization of the asialoglycoprotein receptor in a continuous hepatoma line. *J Biol Chem* **256**: 8878–8881

Torrani Cerenzia M, Fiume L, De Bernardi Venon W, Lavezzo B, Brunetto MR, Ponzetto A, Di Stefano G, Busi C, Mattioli A, Gervasi GB, Bonino F and Verme G (1996) Adenine arabinoside monophosphate coupled to lactosaminated human albumin administered for 4 weeks in patients with chronic type B hepatitis decreased viremia without producing significant side effects. *Hepatology* **23**: 657–661

Trerè D, Farabegoli F, Cancellieri A, Ceccarelli C, Eusebi V and Derenzini M (1991) Ag-NOR protein quantity in human tumours correlates with the proliferative activity evaluated by bromodeoxyuridine labeling and Ki-67 immunostaining. *J Pathol* **165**: 53–59

© 1999 Cancer Research Campaign
